# Sex in Education

**Published:** 1874-10

**Authors:** 


### SEX IN EDUCATION.

Dr. Clark's book has been abused, ridiculed and contemned, but it has never been answered, and for the simple reason it is irrefragable; it is founded on a rock so substantial, so built in the physiological constitution, that you might as well attempt to remove the sun from its place. When he says that the co-education of the sexes is not rational, is injurious, he means that our sons and daughters, from ten to twenty-five, ought not to study in the same classes, because the girls cannot keep up with the boys without such an injury to the physical constitution as in the end would destroy the race. He does not say that a woman cannot study as much as a man; that her intellect is inferior; that her capabilities, her capacities are not as extensive; he does not claim that a woman cannot be as able as a physician, clergyman, lawyer, editor, astronomer or mathematician as a man, but that if she studies hard enough thus to become, she makes an unnatural use of herself, she thwarts the Almighty in the great purposes of her existence.

Every created thing was made for some main, leading, specific purpose, and the perfection of that thing, having life, can only be secured by cultivation, by training, by nurture, by practice in that thing, by being educated up to it. Woman was made to perpetuate the race, to people the globe, and to fill heaven with redeemed immortals, and she has been created with instincts and feelings and affections wisely and kindly adapted to that end, physically and morally; to pervert these to other aims is always unwise, unsafe and unholy. Before the child is born the mother's nature goes out towards it with yearnings unutterable; she begins to make its little dresses with a luscious interest which no pen can describe; when it comes into the world she looks upon it with a bliss that an angel well might envy, and from that hour her whole existence is absorbed in its care, a care which never sleeps, for in her dreams she lives in her little one; and all this is necessary, essentially necessary, for of all that is born into the world the human child is the most hopelessly helpless, and that care should never cease until marriage or maturity. Shall that mother wrench and pervert and blight and blast her whole nature by leaving her infant to the unloving care of stranger hands, of purchased attentions, and go and cut off some man's leg, or preach a sermon on the doctrine of election, or deliver a lecture on the indestructibility of matter, or write a leader on the philosophy of free trade?

The true elevation of the race is in making men more manly; in educating women to be more womanly; whatever reverses that order perverts the end of human existence. Our very instincts are shocked at the sight of a masculine woman or an effeminate man; we despise the man, we shrink from the woman, as a kind of monster; hence to educate them alike and together is an absurdity. It is useless to deny that women were made to have children, and every one of them ought to have a chance. The man was made to support his wife, to take care of her, to protect her, to provide for her and for his family, and he who does not take a wife to himself does not answer the end of his creation, is a drone, ought to be hustled out of the human hive and sent off to some place of penal servitude for the inglorious remnant of his natural life. Old bachelors are of no account anyhow; their whole lives are a failure; they are the ones who have nothing to show for all their labor under the sun but wood, hay, stubble, and hence, for all the good they have done in the world, they had better never have been born.

All wake up in the morning with a certain amount of nervous energy, to be expended during the day on the various parts of the body according to their requirements, to the brain of a student to the muscles of a workman; if the requisite amount is not sent to the brain, the man cannot think easily; if the muscles are not fully supplied, work is a labor, an effort, a failure. For one week in every month from puberty to the end of child-bearing, the reproductive organs are in a state of excitement of development, during which an excess of nervous power is essentially necessary, in order to the proper performance of the functional requirements; to deny this is idiotic. If, during that week the female is required to study as hard as the male, or work as hard as the male, then the reproductive system is to that extent deprived of its natural proportion of nervous energy, and must to that extent suffer, by being imperfectly developed, to the inevitable injury, moral, mental, and physical, of the being born thereafter; and this being continued from generation to generation, there comes a point when nature refuses to produce at all, just as the time comes to a fruit tree when it has no power to blossom, or if at all, it is so imperfect as not to fructify.

It is true that working women have more and stronger children than those who do not work; that slaves propagate faster than their masters, but no one can expect that the race will be elevated to a very high point if it remains in a condition of servitude, or menial manual labor; besides, in discussing the subject of the co-education of the sexes, it is the mental co-education, the schooling, which is under consideration, the mental and moral, and not the muscular view of the case.

It is not to the point to be able to name an occasional case of a woman who has obtained a man's reputation in any branch of science or art, for these are exceptional cases; nor is it pertinent to say that in girls' schools the general health is quite as good as among academicians and college students, not only because the competition in the same class does not exist, but the ill effects are gradual, and often so imperceptible in their growth as not to be perceived until the education is completed, when many a time they lay down and die even within a month after graduation; sometimes they keep up until marriage, when premature births, miscarriages, deformities, and other misfortunes, too often tell the tale of the great mistake in the mode of education.

If the race is to be perfected, there must be a philosophical attention to the reproductive system and seasons, which should be seasons of rest, mental and physical; the "period" is on trial, and its whole duration, whether three days or a week, should be spent in quietude, beginning at the twelfth year, when nothing should be done which requires any great exertion of mind or body; there should be sweet repose of both, only enough thinking or study, or work to prevent a feeling of restlessness or despondency, including three or four, or five hours every day in open air activities, such as may be brought out in riding, walking, visiting, and lively wayside conversation, so as to send as much strength as possible to the reproductive organs. In succinct phrase, cultivate all that is feminine in woman, cultivate all that is masculine in man; that is masculine which pertains to business and affairs ; that is womanly which pertains to children and the fireside, in short to domestic life, and whatever is beyond this is a mistake, a misfortune, a monstrosity, and a crime ; a crime against society, humanity, and the Infinite One.

